# Convergent Evolution of Antibiotic Tolerance in Patients with Persistent Methicillin-Resistant Staphylococcus aureus Bacteremia

**DOI:** 10.1128/iai.00001-22

**Published:** 2022-03-14

**Authors:** Mitra M. Elgrail, Edwin Chen, Marla G. Shaffer, Vatsala Srinivasa, Marissa P. Griffith, Mustapha M. Mustapha, Ryan K. Shields, Daria Van Tyne, Matthew J. Culyba

**Affiliations:** a Department of Medicine, Division of Infectious Diseases, University of Pittsburgh School of Medicine, Pittsburgh, Pennsylvania, USA; b Center for Evolutionary Biology and Medicine, University of Pittsburgh School of Medicine, Pittsburgh, Pennsylvania, USA; New York University School of Medicine

**Keywords:** antibiotic tolerance, MRSA, within-host evolution, tricarboxylic acid cycle, stringent response

## Abstract

Severe infections caused by methicillin-resistant Staphylococcus aureus (MRSA) are often complicated by persistent bacteremia (PB) despite active antibiotic therapy. Antibiotic resistance rarely contributes to MRSA-PB, suggesting an important role for antibiotic tolerance pathways. To identify bacterial factors associated with PB, we sequenced the whole genomes of 206 MRSA isolates derived from 20 patients with PB and looked for genetic signatures of adaptive within-host evolution. We found that genes involved in the tricarboxylic acid cycle (*citZ* and *odhA*) and stringent response (*rel*) bore repeated, independent, protein-altering mutations across multiple infections, indicative of convergent evolution. Both pathways have been linked previously to antibiotic tolerance. Mutations in *citZ* were identified most frequently, and further study showed they caused antibiotic tolerance through the loss of citrate synthase activity. Isolates harboring mutant alleles (*citZ*, *odhA*, and *rel*) were sampled at a low frequency from each patient but were detected in 10 (50%) of the patients. These results suggest that subpopulations of antibiotic-tolerant mutants emerge commonly during MRSA-PB. Methicillin-resistant Staphylococcus aureus (MRSA) is a leading cause of hospital-acquired infection. In severe cases, bacteria invade the bloodstream and cause bacteremia, a condition associated with high mortality. We analyzed the genomes of serial MRSA isolates derived from patients with bacteremia that persisted through active antibiotic therapy and found a frequent evolution of pathways leading to antibiotic tolerance. Antibiotic tolerance is distinct from antibiotic resistance, and the role of tolerance in clinical failure of antibiotic therapy is defined poorly. Our results show genetic evidence that perturbation of specific metabolic pathways plays an important role in the ability of MRSA to evade antibiotics during severe infection.

## INTRODUCTION

Severe infections caused by methicillin-resistant Staphylococcus aureus (MRSA) are often complicated by persistent bacteremia (PB) (1). PB occurs when patient blood samples remain culture positive for several days to weeks despite active antibiotic therapy. During treatment, bacteria are killed primarily by the combination of antibiotics and host phagocytic cells (2). PB represents inadequate control of the infection and is associated with increased mortality (3–6). Vancomycin, daptomycin, and ceftaroline are the current mainstays of antibiotic therapy for severe MRSA infections. MRSA evolves antibiotic resistance only rarely during therapy (1). This information argues that the primary bacterial mechanisms contributing to PB are antibiotic tolerance and host immune evasion, rather than antibiotic resistance.

Antibiotic tolerance is distinct from antibiotic resistance. Resistance occurs when a bacterial strain can grow in the presence of an antibiotic concentration that typically halts its growth, as evidenced by a higher MIC of the antibiotic. Resistance is conferred by mutations or by the acquisition of resistance genes that interrupt the drug-target interaction and are typically specific to one antibiotic class (7). Tolerance, by contrast, is a measure of bacterial survival and manifests as decreased antibiotic killing (8). Mutations that cause tolerance phenotypes are not specific to the drug-target interaction. Instead, they perturb metabolic functions in the cell, which slows antibiotic target turnover, preventing killing by multiple antibiotic classes (9, 10). For example, although isolated infrequently, small colony variants (SCVs) of Staphylococcus aureus display a strong association with persistent S. aureus infections and harbor mutations that cause slow growth and antibiotic tolerance (11). Antibiotic tolerance can also occur independently of mutation, either via gene regulation (12) or stochastic gene expression (13). Our mechanistic understanding of antibiotic tolerance is due largely to characterizing mutations that have been found to enhance or diminish it. Due to its plastic nature, tolerance is difficult to study in clinical settings and tolerance mutations are thought to be rare.

S. aureus PB can be considered a natural experiment in microbial evolution. The infecting population is large enough to contain significant genetic diversity due to spontaneous mutation and is also subject to strong selection within the host environment (14). Within-host evolution can be tracked by analyzing serial culture-positive blood samples with whole-genome sequencing (WGS). This process is often accomplished using a “phenotype-first” approach. For example, the within-host evolution of SCVs and antibiotic resistance has been tracked by first recognizing the phenotype in the clinical microbiology lab and then using WGS to compare the genomes of bacterial isolates with and without the phenotype to identify candidate causal mutations (15). However, not all tolerance mutations lead to overt growth defects (16), and the SCV phenotype is encountered rarely (11); thus, this phenotype-first approach is likely to miss tolerance mutations.

To address this limitation, we used WGS to track the within-host evolution of MRSA in patients with PB, without preselecting for an SCV or antibiotic resistance phenotype, and analyzed the WGS data for signatures of adaptation. Then, we assessed the contribution of individual mutations to antibiotic tolerance using phenotypic assays. Using this “genotype-first” approach, we found strong evidence for convergent evolution of tricarboxylic acid cycle (TCA) and stringent response genes, two pathways with known links to antibiotic tolerance (13, 16–18). Further study of the mutations we identified in *citZ*, a TCA cycle gene encoding citrate synthase (CS), revealed they cause loss of function and antibiotic tolerance. These mutant alleles remained at low frequency during the infections but were detected in 10 (50%) of the patients. This finding suggests that subpopulations of antibiotic-tolerant mutants emerge commonly during MRSA-PB.

## RESULTS

### Convergent evolution of specific genetic loci during persistent MRSA bacteremia.

**FIG 1:**
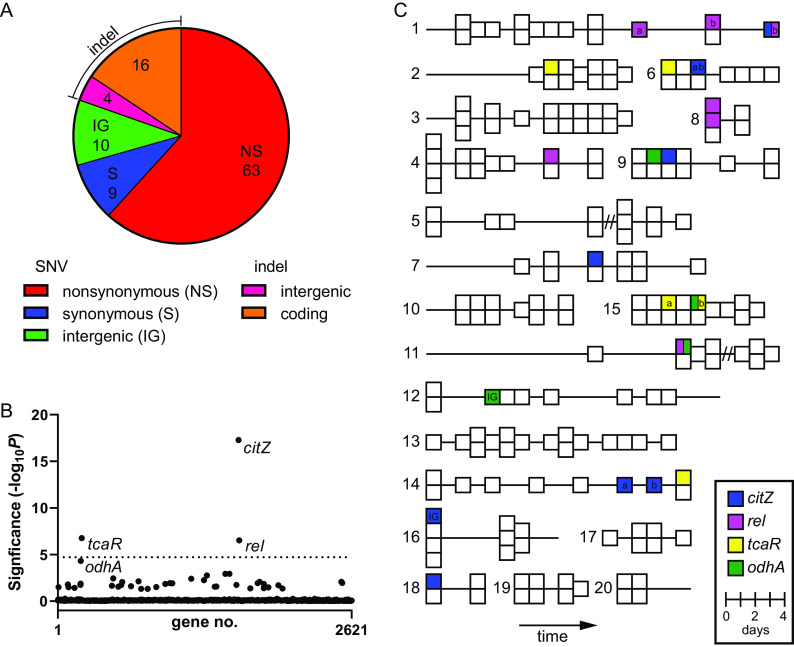
Gene-level enrichment of mutations across different patients. (A) Distribution of mutation types. The area of each slice of the pie chart is proportional to the number of mutations for that mutation type (total *n* = 102). Mutation types are given in the legend. (B) Gene enrichment analysis. Significance of mutation enrichment for 2,621 individual genes. Only nonsynonymous mutations were included. Genes that approach or exceed a Bonferroni-corrected significance threshold of α = 0.05 (horizontal dotted line) are named. (C) Patient plots of PB isolates. Each block represents one of the 206 PB isolates. Isolates are arranged chronologically (from left to right) for each of the 20 patients (patient no. indicated to left of each plot). Isolates from the same day are stacked vertically. Horizontal lines indicate the total duration of bacteremia. Enriched mutant genes from B (*citZ*, *rel*, *tcaR*, and *odhA*) are mapped onto PB isolates, as indicated by the different block colors (see legend). Blocks split with two colors indicate that both mutations were detected in that isolate. Blocks annotated with a or b indicate different mutant alleles of that gene from the same patient. IG, indicates an intergenic mutation upstream of the gene; //, indicates the interval between a resolution and relapse of bacteremia.

To examine within-host evolution using a genotype-first approach, we collected a total of 206 blood culture isolates from 20 patients (∼10 isolates per patient) with MRSA-PB, favoring patients with more prolonged episodes of bacteremia without SCV phenotypes or resistance to the treatment antibiotic. The median duration of bacteremia was 11 days and ranged from 3 to 24 days. As expected, patients were older (median age, 65 years) with multiple medical comorbidities and a variety of different sites of infection (see Table S1 in the supplemental material). The most common anti-MRSA drugs used were vancomycin, daptomycin, and ceftaroline, and every patient received at least one of these agents. We performed WGS on all 206 PB blood culture isolates and compared the genome sequences of isolates between and within each patient. Isolates belonged to 1 of 4 different S. aureus multilocus sequence types (STs), with ST8 accounting for the majority (13 of 20) of the infections (see Table S2 in the supplemental material). Phylogenetic analysis of the PB isolates, including a set of MRSA genomes derived from a surveillance study within the same hospital system, showed that each patient with PB was infected with a single, unique MRSA strain (see Fig. S1 in the supplemental material). PB isolates from the same patient differed by ≤5 single-nucleotide variants (SNVs), whereas PB isolates from different patients, even within the same ST, differed by >75 SNVs. We next identified the types and genomic locations of variants between isolates sampled from the same patient (see Data Set S1 in the supplemental material). We found a total of 102 unique sequence variants. A total of 79 (77%) mutations were protein altering and 63 of 72 (88%) SNVs found in coding regions were nonsynonymous mutations (Fig. 1A), suggesting some variants may have emerged due to positive selection.

Next, we examined the set of protein-altering mutations for evidence of locus-specific convergent evolution, a signal indicative of adaptation (19, 20). Four genes (*citZ*, *rel*, *tcaR*, and *odhA*) had ≥3 independent mutations (Data Set S1). For statistical rigor, we performed a gene enrichment analysis using a method that accounts for gene length and the average density of nonsynonymous mutations within genes across the data set (20). We found all four genes approached (*odhA*) or exceeded (*citZ*, *rel*, and *tcaR*) the cutoff for statistical enrichment (Fig. 1B). Additionally, examination of the intergenic mutations (Data Set S1) revealed that one occurred 74 bp upstream of *citZ* and another occurred 69 bp upstream of *odhA*, suggesting a possible alteration in promoter function. We then mapped the mutant alleles of these four genes onto temporal plots of each patient’s isolates (Fig. 1C). This analysis revealed two important features. First, each mutant allele was detected only in up to two isolates for each infection and subsequent isolates sampled from the same patient typically lacked the mutation. This observation indicates the bacteria harboring these alleles represent a minor, emergent subpopulation. Second, mutations in the same allele arose independently in different patients. We identified nine mutations in *citZ* across seven different patients, four in *odhA* across four different patients, five in *rel* across four different patients, and five in *tcaR* across four different patients (Fig. 1C). This finding is consistent with convergent evolution of an adaptive trait.

Notably, all four of these genes have been associated previously with antibiotic evasion. *citZ* (also called *gltA*) encodes CS and *odhA* (also called *sucA*) encodes the E1 subunit of the α-ketoglutarate dehydrogenase (αKGD) complex. Both enzymes are part of the TCA cycle, which has been linked previously to antibiotic tolerance *in vitro* through use of S. aureus transposon mutant libraries (13, 16). Rel is the master regulatory enzyme of the stringent response, which is another pathway linked to antibiotic tolerance (18, 21). Last, *tcaR* is part of an operon that modulates glycopeptide resistance in S. aureus (22, 23), and the loss of TcaR function has been shown to enhance biofilm formation through increased expression of the intercellular adhesion (*ica*) locus (24). All four of these patients received the glycopeptide antibiotic vancomycin. In summary, the genotype-first approach strongly linked pathways associated with antibiotic tolerance to MRSA-PB. Remarkably, we detected protein-altering mutations in antibiotic tolerance-associated genes (*citZ*, *odhA*, and *rel*) in 10 (50%) of the patients (Fig. 1C), suggesting emergent subpopulations of antibiotic-tolerant mutants is relatively common in MRSA-PB. We also noted that two isolates harbored both a *rel* mutation and a TCA cycle mutation (Fig. 1C, patients 1 and 11), suggesting a possible epistatic interaction.

We also examined the data set (Data Set S1) for genes harboring protein-altering mutations that did not reach statistical significance in the enrichment analysis but have known links to antibiotic or host evasion in S. aureus. We identified two mutations in *mprF* and two in *gdpP*, which are both implicated in daptomycin resistance (25, 26). Notably, three of these four mutations occurred in patient 11, who was treated with daptomycin and where daptomycin resistance was detected during an episode of relapsed bacteremia (see Data Set S2 in the supplemental material). We also found two mutations in *rpoB*, namely, one in patient 4 and one in patient 8. *rpoB* encodes the β-subunit of RNA polymerase, which is the target of rifampin. Rifampin resistance mutations are known to evolve rapidly in *rpoB* after drug exposure (27, 28), and resistance was detected in isolates from both patients (Data Set S2). These patients were not exposed to rifampin during the hospital admission. Possibly, the infecting strains were exposed to rifampin previously or the mutations were selected on the basis of vancomycin or cephalosporin exposure (29–31). Additional genes with known links included *arlR*, *mgrA* (32), *clpX* (33, 34), *ftsH* (35), *fusA* (36), *vraS*, *walK*, and *walR* (37). To our knowledge, the remaining ∼40 genes in the data set have unproven links to antibiotic or host evasion and some may represent novel evasion pathways. Alternatively, some of these mutations may not have been selected for and could be insignificant or represent hitchhiker mutations.

### *citZ* mutations cause loss of citrate synthase activity.

We next focused on *citZ*, as it exhibited the strongest signal in the gene enrichment analysis (Fig. 1B). To gain insight into the impact of the eight nonsynonymous mutations on CS activity, we mapped the mutations onto a homologous CS dimer structure from Pyrococcus furiosus (41% amino acid sequence identity) (38) (see Fig. S2 in the supplemental material). We first noted that none of the mutations mapped to amino acids that are known to be involved directly in substrate binding or catalysis (38). The following four mutations mapped to CS dimer interfaces: two (S201P and A210V) localized to a large central dimerization interface comprised of multiple α-helices and two (G7D and P354S) were located on packing antiparallel strands alongside the exterior of the dimer. CS is a highly conserved functional homodimer, and each monomer contributes key amino acids to the substrate binding site of its pair. Dissolution of the homodimer into structurally intact monomers renders the enzyme inactive (39); thus, the location of the above four mutations predicted impairment of enzyme activity. Three other mutations (A313P, A313V, and V315D) mapped to packing α-helices within the interior structure of the CS monomer. The location of these mutations predicted destabilization of the hydrophobic core. Finally, the last mutation we identified (D141N) mapped to a solvent-exposed flexible loop distant from the enzyme’s functional sites. However, D141N co-occurred with G7D, so the protein was still predicted to be disrupted through the G7D mutation.

**FIG 2:**
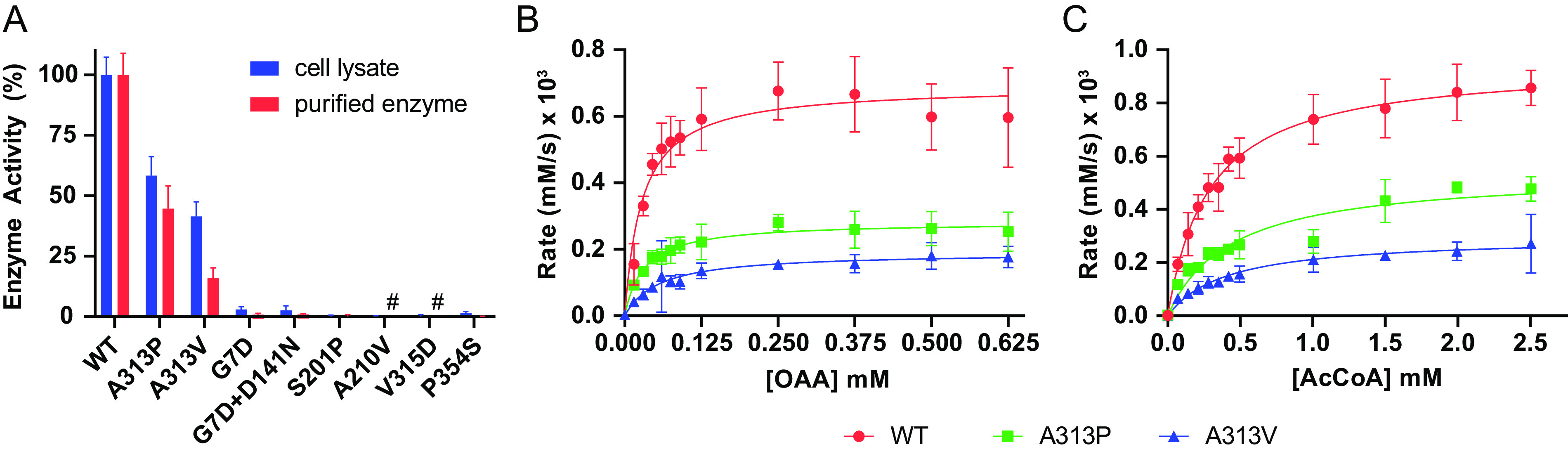
*citZ* mutations cause a loss of citrate synthase activity. (A) Percent citrate synthase activity. CS rates were measured using the lysate of each clinical isolate harboring a *citZ* mutation (blue) and the corresponding purified recombinant CS protein containing the same mutation (red). Rate values are normalized to respective wild-type (100%) and buffer-only controls (0%). Data points and error bars represent the mean and 95% confidence intervals of at least three independent replicates, respectively. #, denotes mutants with no purified enzyme data due to poor expression. (B and C) Plots of CS rate versus substrate concentration for wild-type (WT) and A313 mutants (A313P and A313V). (B) Variable oxaloacetic acid (OAA) with acetyl coenzyme A (AcCoA) fixed at 0.3 mM. (C) Variable AcCoA with OAA fixed at 0.5 mM. Data points and error bars represent the mean and 95% confidence intervals of independent replicates (*n* = 3), respectively. Plotted curves indicate best fits from nonlinear regression using the Michaelis-Menten equation. See Table S3 for values of fitted parameters (*k*_cat_ and *K_m_*).

To understand the functional impact of the mutations, we assayed enzyme activity in cell lysates of bacterial isolates harboring *citZ* mutations and purified recombinant versions of the enzymes. As predicted by the structural analysis above, *citZ* mutations caused significantly reduced or a complete loss of CS activity (Fig. 2). Two recombinant mutant proteins (A210V and V315D) did not express well and could not be purified, which is indicative of protein instability leading to loss of function. The remaining proteins expressed and purified similarly to wild-type CS, suggesting proper folding. Measurement of the CS activity from these recombinant enzymes correlated well with CS activity from the lysates (Fig. 2A), further supporting that the CS activity defects we observed in cell lysates were due specifically to altered CS function. The recombinant D141N enzyme displayed wild-type activity, consistent with its location on the crystal structure. However, this mutation co-occurred with G7D in the clinical isolate, and both the cell lysate and the recombinant enzyme that contained both mutations (D141N+G7D) displayed a loss of CS activity, which was similar to G7D alone.

Two of the recombinant CS mutants (A313P and A313V) retained enough activity to investigate their mechanism by kinetic analysis. To do this analysis, we examined the rate of product formation as a function of substrate concentration and then fit the data to a Michaelis-Menten kinetic model (Fig. 2B and C). CS has two substrates, namely, oxaloacetic acid (OAA) and acetyl coenzyme A (AcCoA). In one set of experiments, we varied the OAA concentration with a fixed AcCoA concentration (Fig. 2B), and in another set, we varied the AcCoA concentration with a fixed OAA concentration (Fig. 2C). In both cases, we found the mutations caused a significant increase in *K_m_* and a significant decrease in *k_cat_* (see Table S3 in the supplemental material), suggesting the mutations disrupt both substrate binding and catalysis. In summary, our biochemical analysis demonstrates that the *citZ* mutations that arose during MRSA-PB impair CS activity by disrupting the overall structure of the enzyme.

### *citZ* mutations cause antibiotic tolerance.

**FIG 3:**
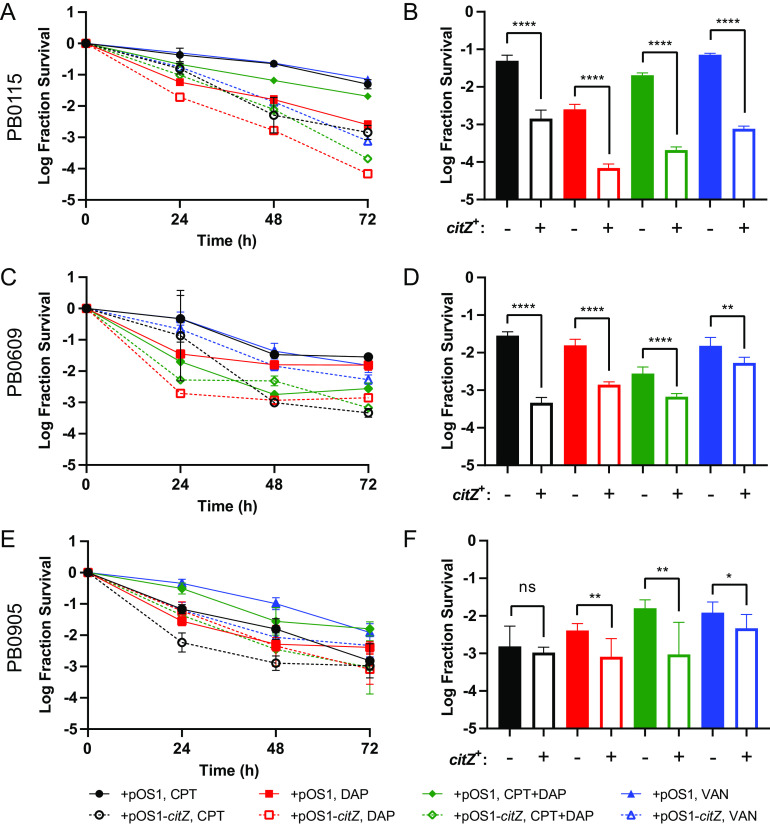
Effect of *citZ* mutations on antibiotic killing. Time-kill curves of clinical *citZ* mutants PB0115 (A), PB0609 (C), and PB0905 (E) complemented with empty vector (pOS1, solid lines) or wild-type *citZ* (pOS1-*citZ*, dashed lines) exposed to ceftaroline (CPT, black), daptomycin (DAP, red), ceftaroline + daptomycin (CPT+DAP, green), or vancomycin (VAN, blue). Fraction survival at the 72-h time point is replotted for PB0115 (B), PB0609 (D), and PB0905 (F) to aid the comparison. Data points and error bars represent the mean and 95% confidence intervals of independent replicates (*n* = 3 to 6), respectively. Mean values were compared using a two-tailed *t* test (ns, *P* > 0.05; *, *P* < 0.05; **, *P* < 0.01; ****, *P* < 0.0001).

TCA cycle defects have been shown previously to cause antibiotic tolerance in laboratory strains of methicillin-sensitive S. aureus (MSSA) (13, 16). Therefore, we hypothesized that the *citZ* mutations we identified in patients with MRSA-PB would also cause antibiotic tolerance. To test this hypothesis, we complemented three of the clinical isolates harboring loss-of-function *citZ* mutations with either wild-type *citZ* (pOS1-*citZ*) or empty vector (pOS1) and then measured bacterial survival through time after exposure to ceftaroline, daptomycin, vancomycin, or the combination of daptomycin plus ceftaroline (Fig. 3). These antibiotics were chosen for their clinical relevance in the treatment of severe MRSA infection. Notably, all three isolates were derived from a patient exposed to at least one of these antibiotics (Table S1). We observed more rapid antibiotic killing upon restoration of wild-type *citZ* (*citZ*^+^) for all three isolates (Fig. 3A, C, and E). In general, a significant increase in survival due to *citZ* mutation was sustained up to 72 h for all antibiotic exposures (Fig. 3B, D, and F), with the only exception being isolate PB0905 during ceftaroline challenge (Fig. 3F). We also noted that the combination of daptomycin plus ceftaroline did not overcome the tolerance effect of the *citZ* mutations. Importantly, the effect of the *citZ* mutations was specific to the rate of antibiotic killing, as *citZ*^+^ complementation did not significantly affect growth rates or MICs (see Table S4 in the supplemental material). We also obtained similar results in control experiments using a *citZ* transposon insertion mutant of MRSA strain JE2 compared with the parental JE2 strain (40) (see Fig. S3 in the supplemental material). Last, we measured growth rates and MICs to five different antibiotics for clinical isolates harboring TCA cycle or *rel* mutations and compared them with control isolates derived from the same patient that did not harbor these mutations (see Data Set S3 in the supplemental material). An analysis of these data revealed that mutations in *rel*, and not the TCA cycle genes (*citZ*/*odhA*), were associated with a growth defect (Fig. S4A and B). Neither set of mutant alleles displayed an association with resistance (Fig. S4C and D). We conclude that the within-host evolution of *citZ* mutations causing a loss of CS function leads to a multidrug antibiotic tolerance phenotype in MRSA without an overt growth defect.

## DISCUSSION

We used a genotype-first approach to study the within-host evolution of MRSA during clinical cases of PB and repeatedly observed the evolution of antibiotic tolerance through dysregulation of the TCA cycle. TCA cycle mutations have been linked previously to antibiotic tolerance *in vitro* using laboratory strains of S. aureus (13, 16). However, they have not been consistently observed in clinical cases of S. aureus bacteremia analyzed by WGS. We suspect this discrepancy is due to two main factors. First, many prior studies have used a phenotype-first approach, for example, by focusing on patients that evolved isolates with resistance to the treatment antibiotic (37, 41). Antibiotic resistance and tolerance are mechanistically distinct (8), so it is not surprising that tolerance mutations would not be found in studies that preselected isolates for phenotypic resistance. Second, many prior studies did not focus on PB or only sampled a single blood isolate per patient (20, 42–44). In contrast, we focused on patients with more prolonged courses of PB (median duration, 11 days), sampled an average of 10 blood isolates per patient, and found that the mutants represented a minor subpopulation in the blood. Therefore, we suspect prior studies of PB did not have the sampling depth to detect these minor subpopulations. Future studies of PB may achieve even greater sensitivity by sequencing the entire population of bacteria captured in blood cultures, as opposed to sequencing only a single isolate from each culture (14).

MRSA colonizes human skin, and MRSA infections result from tissue invasion of this colonizing population (20). One limitation in our data set is that we did not sample the colonizing population. Therefore, it is difficult to ascertain whether the mutations we observed arose prior to or after invasion. Notably, the largest study of within-host evolution of S. aureus to date examined 1,163 clinical isolates and catalogued 1,322 *de novo* mutations that arose within 105 patients (20). The isolates were sampled from both colonizing and infecting sites of the same patients, enabling an assessment of invasion potential. However, gene-level statistical enrichment of TCA cycle genes was not observed and no *rel* mutations were identified. This result was also true of a smaller study that compared paired patient isolates derived from sites of colonization and invasion (43). In contrast to our study, the patients in the above studies were not selected on the basis of PB or sampled serially, so the infections were likely to be milder in severity with less opportunity for the detection of evolution after invasive disease was established. Given our results in a patient cohort with PB, these data are consistent with the idea that evolution of antibiotic tolerance mutations is more common in PB and is driven postinvasion.

We are aware of one prior study of within-host evolution during MRSA-PB that did detect significant enrichment of *citZ* mutations (45). Interestingly, this study focused specifically on nine patients that evolved daptomycin resistance during therapy. Although the most consistent mutations were found to be in phospholipid biosynthesis genes (*mprF*, *cls2*, and *pgsA*), *citZ* mutations were detected in two of the patients. *citZ* is not known to cause daptomycin resistance, and *in vitro* evolution of daptomycin resistance has not been found to produce mutations in *citZ* (45, 46). In our study, two of the patients with *citZ* mutations had no known exposures to daptomycin. Furthermore, none of the 11 isolates with TCA cycle mutations displayed a significant MIC elevation to daptomycin (Fig. S4D), and specific knockout or complementation of *citZ* had no effect on daptomycin MIC (Table S4). Thus, *citZ* mutations alone are not sufficient to cause daptomycin resistance and can be selected for independently of daptomycin exposure. One possible explanation for the high co-occurrence of *citZ* and daptomycin resistance mutations observed previously (45) is that *citZ* mutations could facilitate the evolution of daptomycin resistance mutations via their antibiotic tolerance effect.

Notably, the mutations we identified in *citZ* did not cause an overt growth defect, which is consistent with prior reports (13, 16). This finding differs from the antibiotic tolerance mutations described for SCVs (11), which by definition exhibit slower growth, as well as those described for *rel*, which also cause slower growth (17). Furthermore, the TCA cycle has been shown to be dispensable in a mouse model of osteomyelitis (47). These observations suggest that a loss of TCA cycle function carries relatively little fitness cost during the establishment and maintenance of infection, while simultaneously causing tolerance to antibiotic therapy. This result may be one reason why TCA cycle mutants were isolated frequently in our study. Additionally, biochemical analysis of the *citZ* mutations showed that loss of function was due to global disruption of protein structure. This finding contrasts with, for example, *rel*, where tolerance evolves only via a specific set of mutations that cause partial activation of the stringent response (17) since cells with a fully constitutively activated stringent response are not viable. This response is likely another key factor that explains the high frequency of TCA cycle mutations we observed because, statistically, there are more possible mutations that cause loss of function than gain of function. We were also intrigued that the 11 TCA cycle mutations we identified occurred in only 2 of the 10 steps of the cycle (CS and αKGD) because, *in vitro*, inactivation of the enzymes involved in the early, middle, or late steps of the TCA cycle all cause antibiotic tolerance (13). This information suggests an enrichment for mutations that specifically alter these two steps. Possibly, altering flux through specific steps of the TCA cycle carries an additional advantage *in vivo*. For example, a previous study has shown that citrate can allosterically activate catabolite control protein E (CcpE) in S. aureus and that the loss of CcpE activity results in increased virulence (48). Additional studies are needed to further elucidate the relationship between TCA cycle flux and virulence.

In addition to the TCA cycle genes, we found a significant enrichment of protein-altering mutations in the stringent response regulator *rel*, which is also linked to antibiotic tolerance. The Rel protein of S. aureus (Rel*_Sa_*) is a member of the RelA-SpoT Homologue (RSH) family of enzymes, which regulate the stringent response by controlling intracellular levels of the “alarmone” molecule (p)ppGpp (18). Rel*_Sa_* is a bifunctional RSH enzyme that forms homodimers in solution and regulates (p)ppGpp via modulation of its two opposing (p)ppGpp synthesis and hydrolysis activities (49). The enzyme flips from a net hydrolysis state to a net synthesis state by sensing the presence of an uncharged tRNA on the ribosome (18, 50). The N-terminal half of the protein contains both the hydrolase domain (HD) and synthetase domain (SD), whereas the C-terminal half of the protein consists of several domains important for regulating the SD and HD activities via homodimer formation and tRNA/ribosome interactions. The precise mechanism for how the C-terminal half of the protein interacts with the ribosome in a manner that alters the activities of the HD and SD remains an active area of investigation (18, 49–51). Previously, a clinical *rel* mutant was identified from a patient with MRSA-PB with the SCV phenotype (15) and also from a severely immunosuppressed patient with an unusually prolonged course of Enterococcus faecium PB (52, 53). Both mutations were located in the HD and were shown to promote antibiotic tolerance by increasing (p)ppGpp levels and partially activating the stringent response (17). Here, we bolster these findings by uncovering five additional novel *rel* mutations derived from four different patients, including four mutations that lie outside the HD. Based on the locations of these mutations, we can speculate on their functional effects. D141Y is located in the HD and is likely to reduce hydrolase activity resulting in a net elevation in (p)ppGpp levels, possibly similar to the two previously described clinical *rel* mutations (17). The effects of the remaining four mutations are less certain but presumably also disrupt the regulation of the stringent response. A308T is located in the SD, so it may alter synthetase activity. E391K is located on a linker region between the N- and C-terminal halves of the protein, and the last two mutations (V677G and a frameshift at codon 704) are located within the C-terminal ACT domain, which interacts with the ribosome and also contributes to Rel homodimerization (49–51). These latter mutations are likely to perturb HD/SD activity by altering regulatory interactions with the ribosome, possibly by disrupting homodimerization. Biochemical characterization is needed to understand these mutations and may provide further insights on the regulatory mechanism of Rel*_Sa_*.

The mutant bacteria we detected represent only a subpopulation within each infection. However, their antibiotic tolerance mutations likely represent the extreme, genetically fixed case of a plastic phenotype that varies within the entire infecting bacterial population. This variation is because antibiotic tolerance can be induced by the environment independently of DNA coding changes via gene regulation. For example, in the case of *rel*, antibiotic exposure and nutritional stress are potent activators of Rel’s (p)ppGpp synthesis activity, which induces the stringent response and leads to a multidrug antibiotic tolerance phenotype (18). Antibiotic tolerance can also result from stochastic gene expression, and it has been shown that low expressers of TCA cycle genes within bacterial populations are antibiotic tolerant (13). Additionally, oxidative stress may reduce TCA cycle flux through destruction of the iron-sulfur clusters of key TCA cycle enzymes (54), causing antibiotic tolerance independently of any DNA coding changes. Furthermore, low TCA cycle flux is associated with a reduction in the proton motive force (PMF) across bacterial membranes, and some antibiotics depend on PMF for entry into cells in order to be active (55). Antibiotic tolerance can also be induced within phagocytic cells that have internalized S. aureus (54, 56), and these internalized bacteria may represent a reservoir for relapsing and persistent S. aureus infections. Possibly, the TCA cycle and *rel* mutations we detected evolved within the phagosomes of host immune cells. Ultimately, the specific selective pressures and antibiotic classes that drive the emergence of tolerant mutants in *in vivo* are unknown, and both *in vitro* and animal model studies will be necessary to elucidate these mechanisms.

Finally, the emergence of highly drug-tolerant subpopulations is clinically concerning, as it may hasten the acquisition of frank resistance mutations in patients with PB (57). Adding metabolites to culture media that bypass the TCA cycle (10, 55), or inhibiting Rel activity (58–61), can reverse the tolerance phenotype, suggesting it may be possible to target tolerance for therapeutic benefit. There is a need for better therapies to eradicate persistent MRSA infections. Our *in vivo* findings, derived from patients with PB, support the idea that potent antitolerance drugs could serve as adjuvants that enhance the antibiotic activity of traditional antibiotics.

## MATERIALS AND METHODS

### Bacterial sampling and DNA extraction.

Patients admitted to the University of Pittsburgh Medical Center with MRSA-PB were identified by consecutive dates of positive blood cultures over a period spanning from March 2016 to April 2018. For every blood culture ordered for the patient that resulted in at least one positive blood culture bottle, a well-isolated colony from the blood agar plate was sampled. Cultures of the isolates were archived in 15% glycerol at −80°C. Genomic DNA was purified from overnight lysogeny broth (LB) cultures of the individual bacterial isolates using the DNeasy blood and tissue kit (Qiagen). To facilitate bacterial lysis of S. aureus, the extraction protocol was modified by adding lysostaphin (Sigma). DNA was quantified by a NanoDrop instrument (Thermo Scientific).

### Whole-genome sequencing.

Illumina DNA sequencing libraries were constructed using Nextera DNA kits (Illumina) with each isolate assigned a unique barcode. Sequencing was carried out on the NextSeq 550 platform (Illumina). The average depth of coverage across all isolates was 141x (standard deviation of 48 and range of 51 to 286). To facilitate *de novo* genome assembly, one isolate from each infection was sequenced using the MinION platform (Oxford Nanopore Technologies). MinION libraries were prepared using a rapid barcoding kit (SQK-RBK004) and sequenced on R9.4.1 flow cells. Albacore v2.3.3 or Guppy v2.3.1 were used for the base-calling on raw reads (Oxford Nanopore Technologies). *De novo* hybrid assemblies were generated by combining the Illumina and MinION data from the same isolate (62) via Unicycler v0.4.7 or v0.4.8-beta (63), which resulted in a custom reference genome unique to each infection. Mutations in each bacterial isolate were then identified with *breseq* (64) using the custom hybrid assembly genome derived from the same infection as the reference. Only high-quality mutation calls, as determined by the default parameter settings in *breseq* (>20× coverage and >90% variant read frequency), were included in downstream analysis. Prokka v1.13 (65) was used for annotation.

### Phylogenetic analysis and ancestral genotype assignment.

Core genome maximum likelihood phylogenetic trees were generated from raw Illumina reads using an in-house genomics analysis pipeline, as described previously (66). Briefly, core genome single-nucleotide polymorphisms (cgSNPs) among genomes belonging to the same ST were detected using Snippy v3.1. RAxML v8.2 was used to generate phylogenetic trees under the general time reversible model of nucleotide substitution with a categorical model of rate heterogeneity (67). Phylogenetic trees were visualized on iTOL (68). To determine the most likely temporal direction of evolution for each identified variant, we examined the allele frequencies within each ST, including both the PB sequences and the surveillance study sequences and then assigned the more frequent allele as ancestral.

### Gene enrichment analysis.

To test for statistically significant enrichment of mutations in a particular gene, we used a previously described method, which utilizes a Poisson regression model that considers gene length and the average density of mutations within genes across the data set (20). Only nonsynonymous mutations were included in this analysis. Briefly, given the total number of mutations we observed, we modeled the expected average mutation density in any gene under the null hypothesis, where no gene enrichment occurs. This result was compared with a model of the alternative hypothesis, where the gene of interest permitted a different mutation density than the rest of the genes in the genome. Density parameters were determined in R (69) by maximum likelihood, and *P* values were computed via a likelihood ratio test with one degree of freedom. We analyzed all protein-coding genes that were shared across the sequenced genomes (*n* = 2,621). Pangenomes were constructed with Roary (70) and captured all the nonsynonymous mutations we identified. *P* values were adjusted for multiple comparisons using the Bonferroni correction. The R script, input file (Data Set S4), and further details are provided in the supplemental materials.

### Bacterial growth rates.

Bacterial growth was monitored for 14 h at 37°C in 96-well plates (Corning 3603) by measuring absorbance at 595 nm using an Infinite F200 plate reader (Tecan). Measurements were acquired every 3 minutes with programmed shaking between measurements. Plates were inoculated with a 1,000-fold dilution of an overnight culture in tryptic soy broth (TSB). Maximum growth rates were determined by nonlinear regression using the Gompertz equation (71) in Prism (GraphPad).

### MIC.

MICs were measured in cation-adjusted Mueller-Hinton broth (MHB) supplemented with 50 μg/mL CaCl_2_ (MHB-Ca^2+^) via broth microdilution in 96-well plates with an inoculum of ∼5 × 10^5^ CFU per mL. Antibiotic test concentrations ranged from 0.0625 μg/mL to 32 μg/mL by 2-fold dilutions. Plates were incubated at 37°C for 16 h without shaking, and results were interpreted in accordance with European Committee on Antimicrobial Susceptibility Testing reading guidelines (72). S. aureus ATCC 29213 was used as a quality-control strain.

### Construction of study plasmids.

Oligonucleotides used for plasmid construction are listed in Table S5 in the supplemental material. Turbo Escherichia coli (New England BioLabs) was used for cloning, and Q5 polymerase (New England BioLabs) was used for PCR. LB agar plates containing either 30 μg/mL kanamycin or 100 μg/mL ampicillin were used for selection. For overexpression of S. aureus *citZ* in E. coli, oligonucleotide pair 1 was used to amplify *citZ* from genomic DNA, which was cloned into pET24a (Novagen) using restriction enzymes NheI and NotI (New England BioLabs) to make pET24a-*citZ*. The cloned *citZ* sequence is identical to GenBank accession LT671859, region 1719009 to 1720130. Mutations were introduced by site-directed mutagenesis. For complementation, oligonucleotide pair 10 was used to amplify the *citZ* locus from genomic DNA, which was cloned into pOS1 (73) using restriction enzymes NheI and BamHI (New England BioLabs) to make pOS1-*citZ*. All plasmids were verified through DNA sequencing (GeneWiz).

### Overexpression and purification of S. aureus citrate synthase.

pET24a-*citZ* and its mutant variants were transformed into BL21(DE3) competent E. coli (New England BioLabs) and grown in lysogeny broth (LB) supplemented with 30 μg/mL kanamycin at 37°C shaking overnight. The next day, overnight cultures were diluted into fresh LB with kanamycin and grown at 37°C until an optical density at 595 nm (OD_595_) of 0.5. Then they were induced with 0.1 mM isopropyl β-d-1-thiogalactopyranoside at 30°C for 6 h before harvesting by centrifugation, resuspending in binding buffer (50 mM NaPO_4_ [pH 7.4], 300 mM NaCl, and 10 mM imidazole), and then freezing at −80°C.

For purification, frozen cell pellets were first thawed, lysed through sonication, and centrifuged at 4°C to clarify the soluble fraction. The soluble fraction was then loaded onto HisPur Cobalt resin (ThermoFisher Scientific) that was equilibrated previously using binding buffer (50 mM NaPO_4_ [pH 7.4], 300 mM NaCl, and 10 mM imidazole) and incubated on a rotator overnight at 4°C. The next day, the supernatant was removed, and the beads were washed (50 mM NaPO_4_ [pH 7.4], 300 mM NaCl, and 10 mM imidazole) and eluted (50 mM NaPO_4_ [pH 7.4], 300 mM NaCl, and 200 mM imidazole) using a batch method. Elution fractions that contained the protein of interest as identified on a denaturing SDS-PAGE gel were pooled and purified subsequently by size exclusion chromatography using an ÄKTA purifier (GF75; Cytiva) with buffer 50 mM Tris (pH 7.0) and 100 mM NaCl. Fractions containing the protein of interest were pooled, concentrated using a spin concentrator (Vivaspin; GE Healthcare), and flash-frozen in liquid nitrogen. Protein concentrations were determined by absorbance at 280 nm. Purity was estimated to be >95% by SDS-PAGE.

### Measurement of citrate synthase activity.

Citrate synthase activity was measured using the citrate synthase assay kit (Sigma CS0720) according to the manufacturer’s protocol. Assays were carried out at room temperature in 96-well plates (Corning 3635) using an Infinite F200 multifunction plate reader outfitted with a 412-nm absorbance filter and an injector module (Tecan). Reactions were carried out using either 2 μL of a prepared cell lysate or 2 μL of 0.1 mg/mL purified recombinant citrate synthase (1-μg/mL final concentration). Reactions were initiated by addition of 10 μL of 10 mM oxaloacetic acid (OAA) followed by a programmed 10-s interval of plate shaking. Absorbance readings (412 nm) were collected at 10-s intervals for 90 s. Final reaction conditions were 0.3 mM acetyl coenzyme A (AcCoA), 0.1 mM OAA, 0.1 mM 5,5′-dithiobis-(2-nitrobenzoic acid) (DTNB), and 100 mM Tris-HCl (pH 8.0) in a final volume of 200 μL. For assays that varied the concentration of OAA or AcCoA, the final concentrations of the other reagents were held constant. For measurements of bacterial lysates, cells were pelleted after normalizing overnight culture cell densities and then resuspended in 100 μL of 100 mM Tris-HCl (pH 8.0), 2.5 mM MgCl_2_, and 0.5 mM CaCl_2_. Lysis was carried out by addition of 2 μL of 2.5 mg/mL lysostaphin (Sigma), 5 μL of 5 mg/mL RNase A (Roche), and 5 units of DNase I (New England BioLabs) with incubation at 37°C for 1 h, and then lysates were clarified by centrifugation.

### Genetic complementation of clinical isolates with *citZ*.

Plasmids pOS1 and pOS1-*citZ* were used for complementation. Clinical isolates were confirmed to be inhibited by 10 μg/mL chloramphenicol, enabling selection of pOS1. Electrocompetent cell preparation and transformation were performed essentially as described by Monk et al. (74). Prior to electroporation, pOS1 plasmids were first prepared from E. coli strain IM08B (75) to bypass S. aureus restriction-modification barriers. After electroporation, transformants were selected on TSA plates supplemented with 10 μg/mL chloramphenicol. Single colonies were colony purified once more and screened with colony PCR. The desired insert was then again verified through DNA sequencing (GeneWiz), and the restoration of citrate synthase activity was confirmed by assaying the strain lysate, as described above.

### Antibiotic time-kill assays.

Overnight cultures were diluted 1,000-fold into 2 mL of TSB (Sigma 22092) and grown for 24 h. TSB was supplemented with 10 μg/mL chloramphenicol for the maintenance of plasmid pOS1. A total of 100 μL of culture was removed, serially diluted onto TSA plates, and incubated at 37°C for 18 h to determine the initial population. Cultures were challenged with either with 64 μg/mL (16 to 32× MIC) vancomycin, 32 μg/mL daptomycin (16× MIC), 16 μg/mL ceftaroline (32 to 64× MIC), or the combination of 32 μg/mL daptomycin and 16 μg/mL ceftaroline. After 24 h, 48 h, and 72 h, 100 μL of culture was removed, pelleted, and washed three times with 1 mL of 0.9% saline. To quantify the surviving population, pellets were resuspended in 100 μL of 0.9% saline, serially diluted, plated on TSA, and incubated for 24 h at 37°C prior to colonies being counted. Fraction survival was calculated by dividing the CFU counts of the surviving population by the CFU counts of the initial population.

### Data availability.

The sequences reported here have been deposited with the National Center for Biotechnology Information Sequence Read Archive and GenBank (BioProject accession no. PRJNA773815). GenBank accession no. for the 20 *de novo* assembled-reference genomes can be found in Table S2.
