# Supplementary material for: Convergent Evolution of Antibiotic Tolerance in Patients with Persistent Methicillin-Resistant Staphylococcus aureus Bacteremia
Source: Infect Immun. 2022 Mar 14;90(4):e00001-22. doi: 10.1128/iai.00001-22 (PMC9022596; doi:10.1128/iai.00001-22)
Supplement: SUPPLEMENTAL FILE 5 — Supplemental material. Download iai.00001-22-s0005.pdf, PDF file, 6.2 MB [file iai.00001-22-s0005.pdf]

Supplementary Information

Convergent evolution of antibiotic tolerance in patients with persistent methicillin-resistant *Staphylococcus aureus* bacteremia

Mitra M. Elgrail, Edwin Chen, Marla Shaffer, Vatsala Srinivasa, Marissa Griffith, Mustapha M. Mustapha, Ryan Shields, Daria Van Tyne, Matthew J. Culyba*

*Email: matthew.culyba@pitt.edu

This PDF file includes:

Fig S1. Genomic context of PB isolates.
Fig S2. CS dimer structure with mapped mutations.
Fig S3. Effect of *citZ* Tn insertional activation on antibiotic killing.
Fig S4. Mutant allele association with growth rate and MIC.

Table S1. Clinical characteristics of patients.
Table S2. Patient source, sequence type (ST), and GenBank accession nos. of PB isolates.
Table S3. Michaelis-Menten parameters of wildtype and A313 CS mutants.
Table S4. Growth rates and MICs of pOS1 complemented strains.
Table S5. Oligonucleotides used in this study.

Extended methods: R script for gene enrichment analysis.

SI References

Other supplementary materials for this manuscript include the following:

Dataset S1 (separate file). List of *de novo* mutations detected in PB isolates.
Dataset S2 (separate file). Clinical laboratory antimicrobial susceptibility testing data.
Dataset S3 (separate file). Growth rates and MICs of mutant PB isolates and controls.
Dataset S4 (separate file). R script input file for gene enrichment analysis.

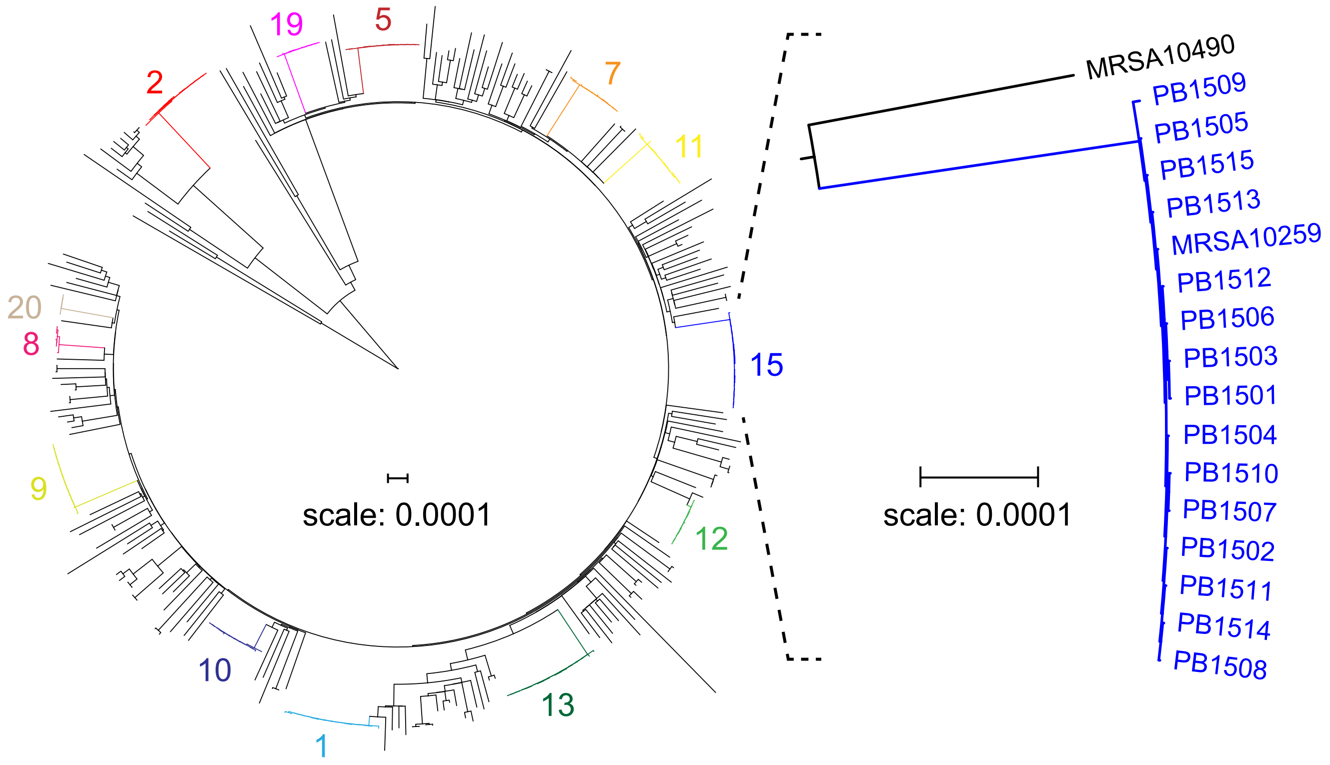

Fig S1. Genomic context of PB isolates. Phylogenetic tree constructed from MRSA ST8 whole genome sequences. Each terminal branch of the tree represents one isolate (see magnified insert of patient 15 branch). Terminal branches/labels shown in color indicate the isolates sampled from the 13 patients with PB due to ST8 (n=136), where each color represents a different patient. Terminal branches/labels shown in black indicate the ST8 isolates sampled from a hospital surveillance study (n=188), where only a single isolate was sequenced from each patient. Branch length is proportional to the fraction of genetic variation due to SNVs. MRSA10259 was verified to be a surveillance study isolate derived from patient 15.

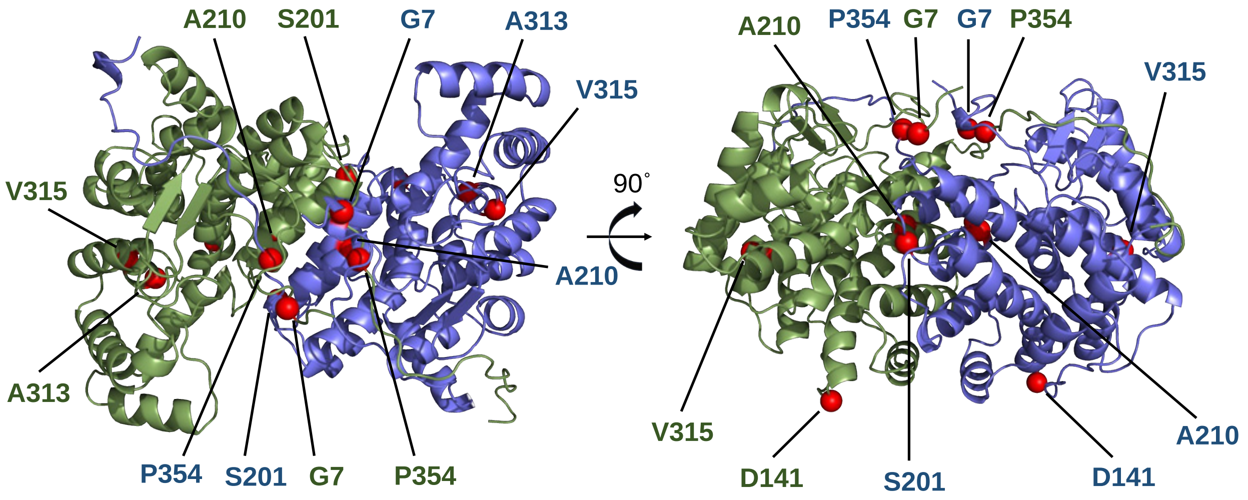

Fig S2. CS dimer structure with mapped mutations. *citZ* mutations identified from clinical isolates are mapped onto a homologous citrate synthase (CS) dimer crystal structure from *P. furiosus* (PDB 1AJ8) (1). CS monomers and their residue labels are colored green and blue. Four mutations map to CS dimer interfaces: two (S201P, A210V) localize to a large central dimerization interface of α-helices and two (G7D, P354S) map to packing anti-parallel strands on the exterior surface of the dimer. Three mutations (A313P, A313V, V315D) map to packing α-helices within the interior structure of the CS monomer. One mutation (D141N) maps to a solvent exposed flexible loop distant from functional sites.

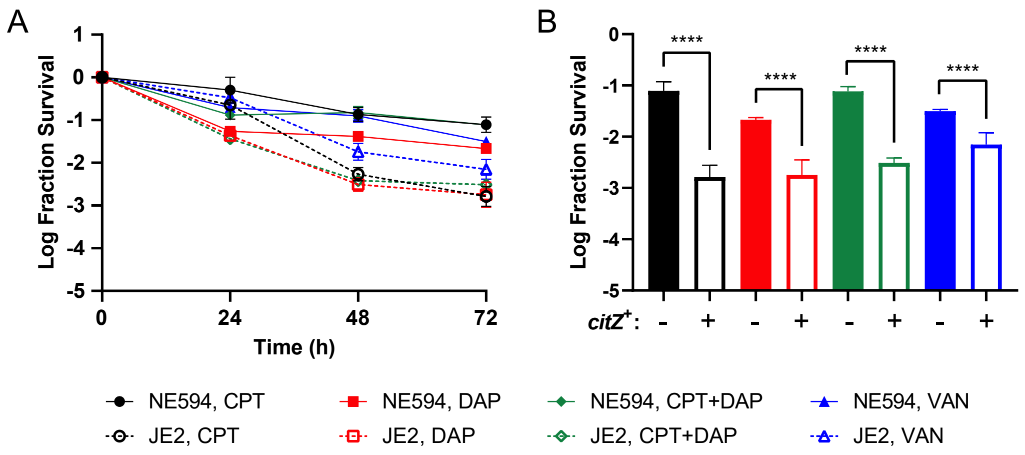

Fig S3. Effect of *citZ* Tn insertional activation on antibiotic killing. (A) Time-kill curves of NE594 and JE2 strains complemented with empty vector (pOS1) exposed to ceftaroline (CPT, black), daptomycin (DAP, red), ceftaroline + daptomycin (CPT+DAP, green), or vancomycin (VAN, blue). (B) Fraction survival at the 72 hour time point is re-plotted to aid comparison. Data points and error bars represent the mean and 95% confidence intervals of independent replicates (n=3-6), respectively. Mean values were compared using a two-tailed t-test (****P<0.0001).

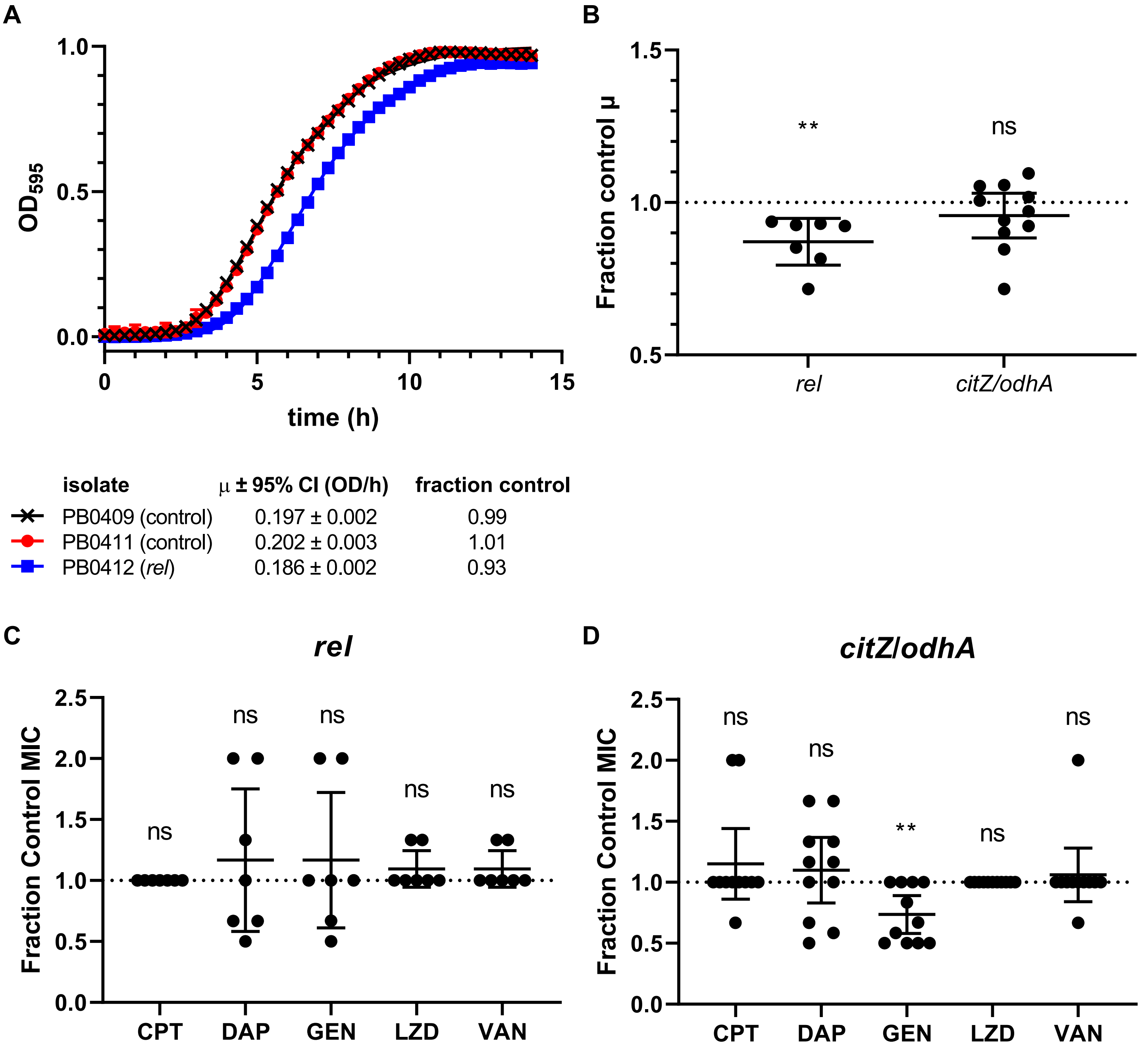

Fig S4. Mutant allele association with growth rate and MIC. (A) Growth curve analysis. The analysis of isolates derived from patient 4 is shown as an example. Isolate PB0412 contains a *rel* frame shift mutation at codon 704 (*rel*, blue squares), which in this case is the only mutation detected relative to PB0409 (control, black X's) and PB0411 (control, red circles). Lines indicate the best-fit from nonlinear regression using the Gompertz equation. Legend: The maximum growth rate (μ) and 95% confidence intervals (95% CI) were determined by nonlinear regression. Values for μ were divided by the average of the two controls (fraction control). (B) Mutant allele association with growth rate. Fraction control μ was determined for isolates containing a protein-coding change in *rel* or TCA cycle genes (*citZ*/*odhA*) and plotted. Mutant allele association with MIC for (C) *rel* and (D) TCA cycle. Fraction control MIC was determined for ceftaroline (CPT), daptomycin (DAP), gentamicin (GEN), linezolid (LZD), and vancomycin (VAN). Lines and error bars represent means and 95% confidence intervals, respectively. Means of *rel* mutants (n=7) and controls (n=7), or TCA cycle mutants (n=11) and controls (n=15), were compared using a two-sided t-test (ns: $P>0.05$; **$P<0.01$). See Dataset S3 for the raw growth rate and MIC data of the isolates.

Table S1

Patient	Age (yr)	Gender	Major co-morbidities	Suspected source of infection	Sites of infection (diagnosis)	Duration of bacteremia (days)	Antibiotic exposures (*anti-MRSA)
1	66	F	DM	chronic skin ulcer	1. heart (endocarditis)<br>2. lung (septic pulmonary emboli)<br>3. spine (discitis/osteomyelitis/abscess)	24	VAN*, DAP*, CFT*, GNT*, TVN*, LZD*<br>PTZ, CIP, MTZ
2	36	F	partial colon transplant, adrenal insufficiency, CHF	central line	1. eye (endophthalmitis)	14	VAN*, DAP*, CFT*<br>CIP
3	75	F	AML, CHF	central line	1. heart (endocarditis)	14	VAN*, DAP*, CFT*<br>MER
4	59	M	DM, PVD	surgical site	1. leg (fem-pop prosthetic bypass graft infection)	12	VAN*, DAP*, CFT*<br>CFZ, PTZ
5	75	F	RA, AF s/p CIED	central line	1. heart (endocarditis)	12 + 5	VAN*, DAP*<br>MTZ, CFX, CFZ, CEF
6	79	F	pancreatic cancer, OA s/ p THR	skin trauma	1. shoulder (septic joint)<br>2. hip (prosthetic joint infection)	8	VAN*, TVN*
7	83	F	heart block s/p CIED, DM, CKD, CHF, venous stasis ulcers, lymphoma	chronic skin ulcer	1. heart (endocarditis)	19	VAN*, LZD*, DAP*<br>CFZ, CIP, PTZ
8	58	M	MI s/p LVAD, h/o MRSA bacteremia with LVAD, CKD, femoral endarterectomy s/p stent	LVAD	1. LVAD hardware and driveline	3	DAP*, CFT*
9	51	M	DM, back pain s/p ACDF, lumbar TLIF	surgical site	1. spine (surgical site infection)	10	VAN*, CFT*<br>MTZ, OXA
10	28	F	IVDU, HCV	IVDU	1. spine (abscess/myositis)<br>2. lung (septic pulmonary emboli)<br>3. hip (osteomyelitis/abscess)	10	VAN*, DAP*, CFT*<br>CEF, PTZ
11	64	M	IDDM, CAD, foot surgical site infection w/ retained hardware	surgical site	1. spine (abscess/osteomyelitis)<br>2. foot (osteomyelitis)<br>3. thumb (osteomyelitis)	20 + 3	VAN*, DAP*, CFT*, LZD*<br>PTZ, CFZ
12	61	M	IVDU, DM, cirrhosis, HCV	IVDU	1. suspected hip<br>2. suspected lung	20	VAN*, GNT*, TVN*
13	61	F	DM, diabetic foot ulcer	chronic skin ulcer	1. foot (osteomyelitis)<br>2. lung (septic pulmonary emboli)<br>3. heart (endocarditis)	17	VAN*, DAP*, CFT*<br>CFZ, PTZ
14	82	F	CVA c/b pressure ulcer, DM, CAD	chronic skin ulcer	1. foot (osteomyelitis)<br>2. heart (endocarditis)<br>3. spine (osteomyelitis<br>4. muscle (abscess)<br>5. brain (embolic stroke)	18	VAN*, DAP*, CFT*<br>AZI, CTX
15	37	F	CHF s/p CIED and home milrinone, DM	central line	1. vascular (infected thrombus)	9	VAN*, DAP*<br>CFZ

Table S1 (cont)

16	81	M	DM, CAD, CVA	occult	Unknown	9	VAN*, DAP*<br>CEF, MTZ
17	80	F	CHF, chronic lymphedema	chronic skin lymphedema	Unknown	6	VAN*, DAP*, CFT*<br>CEF, MTZ, OXA
18	71	M	multiple myeloma	occult	1. spine (discitis/osteomyelitis/abscess)	4	VAN*, DAP*, CFT*<br>PTZ, MTZ
19	69	F	CHF, CVA, Crohn's, RA, COPD	peripheral line	1. shoulder (septic joint)<br>2. hand (septic joint)<br>3. spine (abscess)	5	VAN*<br>CFZ, ASM
20	60	M	COPD, CHF, PSUD	skin trauma	1. lung (pneumonia)	5	VAN*, LZD*, CFT*, RIF*<br>AZI, CEF, PTZ

Table S1. Clinical characteristics of patients. Isolates from a clinical relapse of bacteremia were captured for patients 5 and 11; and the number of days separating the bacteremia events was 55 and 21 days, respectively. The duration of the relapsed bacteremia is listed, separated by '+'. All antibiotic exposures that the patient received during the clinical event are listed.

Abbreviations: female (F), male (M); diabetes mellitus (DM), acute myelogenous leukemia (AML), congestive heart failure (CHF), peripheral vascular disease (PVD), rheumatoid arthritis (RA), atrial fibrillation (AF), status post cardiovascular implantable electronic device (s/p CIED), osteoarthritis (OA), status post left total hip replacement (s/p L THR), chronic kidney disease (CKD), myocardial infarction (MI), status post left ventricular assist device (s/p LVAD), history of methicillin-resistant *Staphylococcus aureus* (h/o MRSA), status post anterior cervical discectomy and fusion (s/p ACDF), transforaminal lumbar interbody fusion (TLIF), intravenous drug use (IVDU), hepatitis C virus (HCV), cerebrovascular accident (CVA), coronary artery disease (CAD), chronic obstructive pulmonary disease (COPD), psychoactive substance use disorder (PSUD); ampicillin-sulbactam (SAM), azithromycin (AZM), cefazolin (CFZ), cefepime (FEP), ceftaroline (CPT), ceftriaxone (CRO), cefuroxime (CXM), ciprofloxacin (CIP), daptomycin (DAP), gentamicin (GEN), linezolid (LZD), meropenem (MEM), metronidazole (MTZ), oxacillin (OXA), piperacillin-tazobactam (TZP), rifampin (RIF), telavancin (TLV), vancomycin (VAN).

Table S2

Patient	MRSA ST	# of isolates	Isolate names	Reference isolate	BioSample accession no.	GenBank accession no.
1	ST8	15	PB0101-PB0115	PB0102	SAMN22513734	JAJHOJ000000000
2	ST8	11	PB0201-PB0211	PB0210	SAMN22513757	JAJHOK000000000
3	ST5	17	PB0301-PB0317	PB0312	SAMN22513770	JAJHOL000000000
4	ST105	14	PB0401-PB0414	PB0401	SAMN22513776	JAJHOM000000000
5	ST8	12	PB0501-PB0512	PB0506	SAMN22513795	JAJHON000000000
6	ST5	10	PB0601-PB0610	PB0601	SAMN22513802	JAJHOO000000000
7	ST8	10	PB0701-PB0710	PB0705	SAMN22513816	JAJHOP000000000
8	ST8	5	PB0801-PB0805	PB0802	SAMN22513823	JAJHOQ000000000
9	ST8	11	PB0901-PB0911	PB0901	SAMN22513827	JAJHOR000000000
10	ST8	11	PB1001-PB1011	PB1005	SAMN22513842	JAJHOS000000000
11	ST8	10	PB1101-PB1110	PB1103	SAMN22513851	JAJHOT000000000
12	ST8	9	PB1201-PB1209	PB1201	SAMN22513859	JAJHOU000000000
13	ST8	16	PB1301-PB1316	PB1313	SAMN22513880	JAJHOV000000000
14	ST5	10	PB1401-PB1410	PB1402	SAMN22513885	CP086215
15	ST8	15	PB1501-PB1515	PB1501	SAMN22513894	JAJHOW000000000
16	ST5	9	PB1601-PB1609	PB1602	SAMN22513910	JAJHOX000000000
17	ST45	6	PB1701-PB1706	PB1704	SAMN22513921	JAJHOY000000000
18	ST105	4	PB1801-PB1804	PB1804	SAMN22513927	JAJHOZ000000000
19	ST8	7	PB1901-PB1907	PB1901	SAMN22513928	JAJHPA000000000
20	ST8	4	PB2001-PB2004	PB2003	SAMN22513937	JAJHPB000000000

Table S2. Patient source, sequence type (ST), and GenBank accession nos. of PB isolates.

Table S3

Enzyme	Variable Substrate	k_{cat} (95% CI) (s^{-1})	K_m (95% CI) (mM)
WT	AcCoA	41 (39 to 43)	0.29 (0.25 to 0.32)
	OAA	30 (28 to 32)	0.03 (0.02 to 0.04)
A313P	AcCoA	23 (21 to 26)	0.42 (0.32 to 0.59)
	OAA	12 (11 to 13)	0.03 (0.02 to 0.04)
A313V	AcCoA	13 (12 to 14)	0.40 (0.32 to 0.49)
	OAA	8 (7 to 9)	0.06 (0.04 to 0.08)

Table S3. Michaelis-Menten parameters of wildtype (WT) and A313 CS mutants. Acetyl coenzyme A (AcCoA), oxaloacetate (OAA), 95% confidence interval (95% CI).

Table S4

Strain	CPT MIC (ug/ml)		DAP MIC (ug/ml)		VAN MIC (ug/ml)		Max growth rate ± 95% CI (OD/h)
JE2+pOS1	0.25	0.25	2	2	4	4	0.211 ± 0.003
NE594+pOS1	0.25	0.25	2	2	2	2	0.234 ± 0.003
PB0115+pOS1	0.5	0.5	2	2	4	4	0.240 ± 0.004
PB0115+pOS1-*citZ*	0.5	0.5	2	2	4	4	0.234 ± 0.004
PB0609+pOS1	0.5	0.5	2	2	2	2	0.283 ± 0.008
PB0609+pOS1-*citZ*	0.5	0.5	2	2	2	2	0.279 ± 0.007
ATCC 29213	0.25	0.25	1	2	2	2	ND

Table S4. Growth rates and MICs of pOS1 complemented strains. Both values from two independent minimum inhibitory concentration (MIC) measurements are shown for ceftaroline (CPT), daptomycin (DAP), and vancomycin (VAN). *S. aureus* ATCC 29213 was used as an MIC assay control. Maximum growth rates and 95% confidence intervals (95% CI) were determined by fitting growth data to the Gompertz equation using nonlinear regression. OD=optical density measured at 595 nm.

Table S5

Pair	Description	Forward Primer (5' to 3')	Reverse Primer (5' to 3')
**1**	*citZ* from gDNA (NheI/NotI)	ACACACGCTAGCATGGCAGAATTACAAAGAGG	GTGTGTGCGGCCGCTTTTCTTTCTTCAAGCGG
**2**	SDM for S201P	GCGGTATCATCATTGCCAGATATGTACTCAG	CTGAGTACATATCTGGCAATGATGATACCGC
**3**	SDM for G7D	CAGAATTACAAAGAGATTTAGAAGGGGTTATC	GATAACCCCTTCTAAATCTCTTTGTAATTCTG
**4**	SDM for D141N	GCTCGAGTAAGACAAAATAAAGAACCACTTAAG	CTTAAGTGGTTCTTTATTTTGTCTTACTCGAGC
**5**	SDM for A313V	GTTGATTTTTATAGTGTGAGTGTTTATCACTG	CAGTGATAAACACTCACACTATAAAAATCAAC
**6**	SDM for A210V	CAGGTATTGTAGCAGTTGTAGGTTCTCTGAAAG	CTTTCAGAGAACCTACAACTGCTACAATACCTG
**7**	SDM for V315D	GATTTTTATAGTGCGAGTGATTATCACTGTATGG	CCATACAGTGATAATCACTCGCACTATAAAAATC
**8**	SDM for A313P	GTTGATTTTTATAGTCCGAGTGTTTATCACTG	CAGTGATAAACACTCGGACTATAAAAATCAAC
**9**	*SDM for P354S	AATTATGCGTAGCAGAGCGAAATATATTG	CTATTATCTTTATATTGTTCTAAAATATG
**10**	*citZ* locus from gDNA (NheI/BamHI)	ACACACGCTAGCATTGTAAAATTCATGG	GTGTGTGGATCCTTATTTTCTTTCTTCAAG
**11**	pOS1 Colony PCR	ATGTTGTGTGGAATTGTGAGC	TAACTTAACTGAAGAACAACG

Table S5. Oligonucleotides used in this study. Primer pairs used for PCR amplification or site-directed mutagenesis (SDM) are listed. * indicates non-overlapping primers were used with the Q5® Site-Directed Mutagenesis Kit (New England Biolabs).

Extended methods: R script for gene enrichment analysis

```
# reads the .csv file into 'mut'
mut <- read.csv(file = 'MyTable.csv')
# runs null hypothesis glm model (all genes have same mutation rate) and assigns output data to 'm0'
m0 <- glm(Mutations ~ offset(log(Length)), family = poisson(link = "log"), mut)
# reads i,j category array header names into 'varlist'
varlist <- names(mut)[-(1:4)]
# creates a blank data frame called 'results' for the forward loop below
results <- data.frame()
# Forward loop: Runs the alternate hypothesis glm model separately for each gene (i), where one gene (i) can have any mutation rate, but all the other genes (j) have the same mutation rate.
# Each output is stored in 'glm.out' and compared via the likelihood ratio test (lrtest) against the null hypothesis ('m0').
# The P-values (Pr) of the lrtest are read into the 'results' data frame.
for(i in 1:length(varlist))
{
fmla <- as.formula(paste0("Mutations ~ ", varlist[i]))
glm.out <- glm(fmla, offset = log(Length), family = poisson(link = "log"), mut)
lrtest.out <- lrtest(m0, glm.out)
Pr <- lrtest.out[2,5]

results[i,1] <- names(coef(glm.out))[2]
results[i,2] <- lrtest(m0, glm.out)[2,5]
}
# writes the P-values of each lrtest into the output file 'MyTable_results.csv'.
# V1 of the output file are the gene names (x1, x2, x3, ..., xn). V2 are the P-values.
write.csv(results, 'MyTable_results.csv')
```

The above R script requires a data input file in .csv format that is named ‘MyTable.csv’ and R packages for running generalized linear models (glm) and likelihood ratio tests. Please see ‘Dataset S4.csv’ as an example for how to format the input file. The first row of the table is a header row. Specific header names are required for the script to function. Each subsequent row represents a gene (open reading frame) in the genome, with all genes represented. The 3rd column of the table must be named ‘Length’ and have a gene length (nt) entered for each gene. The 4th column of the table must be named ‘Mutations’ and have the integer # of mutations entered for each gene. The first two columns of the table can be used to specify gene names or other relevant annotations and are for reference. The table must also include an array of ‘i’ and ‘j’ model category designators for each assigned gene variable (x1, x2, x3, …, xn, where n is equal to the number of genes). This array begins at the 5th column of the table. The column headers for this array must be named ‘x1’, ‘x2’, ‘x3’, …, ‘xn’ for the forward loop in the script to function. Each column in the array represents a specific gene and will have only a single ‘i’ row. The row location of ‘i’ in each column designates the specific gene being tested by the glm. The remainder of the rows will have ‘j’ entered. For example, column ‘x1’ is the array for the 1st gene in the table (row 2). Therefore, ‘i’ is entered in row 2 and all other rows have ‘j’ entered. The purpose of having an array of ‘i’ and ‘j’ category designators for each gene is so the script can use a forward loop to automatically subject each gene separately to the glm while only having to execute the script once in the R command line. The only relevant input data in the table are in the ‘Length’ and ‘Mutations’ columns.

SI References

1. Russell RJ, Ferguson JM, Hough DW, Danson MJ, & Taylor GL (1997) The crystal structure of citrate synthase from the hyperthermophilic archaeon *Pyrococcus furiosus* at 1.9 A resolution. *Biochemistry* 36(33):9983-9994.

Other supplementary materials:

Dataset S1 (separate file). List of *de novo* mutations detected in PB isolates. Protein-altering mutations are grouped by gene and listed in descending order based on the number of independent mutations detected in each gene. The remainder of the list is ordered alphabetically by gene name. Synonymous and intergenic mutations are listed separately below.

Dataset S2 (separate file). Clinical laboratory antimicrobial susceptibility testing data. Values are the minimal inhibitory concentrations (MICs) reported by the clinical laboratory during the patient's hospital stay.

Dataset S3 (separate file). Growth rates and MICs of PB isolates. Growth rates and MICs to the indicated antibiotics were determined for PB isolates containing *citZ*, *relA*, *tcaR*, or *odhA* mutations. 'Control' isolates were selected from the same patient that did not contain these mutations. Listed genes names indicate mutant alleles containing protein-coding changes specific to that isolate relative to the genotype of the control isolates. Values for 'fraction control' were calculated by dividing each raw value by the average value for control isolates derived from the same patient. See Fig S4 for plotted data.

Dataset S4 (separate file). R script input file for gene enrichment analysis.
